# Fecal short chain fatty acids and urinary 3-indoxyl sulfate do not discriminate between patients with Crohn´s disease and ulcerative colitis and are not of diagnostic utility for predicting disease severity

**DOI:** 10.1186/s12944-023-01929-6

**Published:** 2023-10-03

**Authors:** Hauke Christian Tews, Tanja Elger, Stefan Gunawan, Tanja Fererberger, Stefanie Sommersberger, Johanna Loibl, Muriel Huss, Gerhard Liebisch, Martina Müller, Arne Kandulski, Christa Buechler

**Affiliations:** 1 Department of Internal Medicine I, Gastroenterology, Hepatology, Endocrinology, Rheumatology, and Infectious Diseases, University Hospital Regensburg, 93053 Regensburg, Germany; 2 Institute of Clinical Chemistry and Laboratory Medicine, University Hospital Regensburg, 93053 Regensburg, Germany

**Keywords:** Butyrate, Acetate, Calprotectin, Crohn´s disease, Ulcerative colitis, IBD, Biomarker

## Abstract

**Background:**

Urinary 3-indoxyl sulfate levels as well as fecal short chain fatty acid (SCFA) concentrations are surrogate markers for gut microbiota diversity. Patients with inflammatory bowel diseases (IBDs) and patients with primary sclerosing cholangitis (PSC), a disease closely associated with IBD, have decreased microbiome diversity. In this paper, the fecal SCFAs propionate, acetate, butyrate and isobutyrate of patients with IBD and patients with PSC-IBD and urinary 3-indoxyl sulfate of IBD patients were determined to study associations with disease etiology and severity.

**Methods:**

SCFA levels in feces of 64 IBD patients and 20 PSC-IBD patients were quantified by liquid chromatography with tandem mass spectrometry (LC–MS/MS). Urinary 3-indoxyl sulfate levels of 45 of these IBD patients were analysed by means of reversed-phase liquid chromatography-electrospray ionization-tandem mass spectrometry. Feces of 17 healthy controls and urine of 13 of these controls were analyzed in parallel. These cohorts had comparable sex distribution and age.

**Results:**

Urinary 3-indoxyl sulfate concentrations (normalized to urinary creatinine levels) was increased (*P* = 0.030) and fecal isobutyrate levels (normalized to dry weight of the stool sample) of IBD patients were decreased (*P* = 0.035) in comparison to healthy controls. None of the analyzed metabolites differed between patients with Crohn´s disease (CD) and patients with ulcerative colitis (UC). Fecal acetate and butyrate positively correlated with fecal calprotectin (*P* = 0.040 and *P* = 0.005, respectively) and serum C-reactive protein (*P* = 0.024 and *P* = 0.025, respectively) in UC but not CD patients. UC patients with fecal calprotectin levels above 150 µg/g, indicating intestinal inflammatory activity, had higher fecal acetate (*P* = 0.016), butyrate (*P* = 0.007) and propionate (*P* = 0.046) in comparison to patients with fecal calprotectin levels < 50 µg/g. Fecal SCFA levels of PSC-IBD and IBD patients were comparable.

**Conclusions:**

Current findings suggest that analysis of urinary 3-indoxyl-sulfate as well as fecal SCFAs has no diagnostic value for IBD and PSC-IBD diagnosis or monitoring of disease severity.

**Supplementary Information:**

The online version contains supplementary material available at 10.1186/s12944-023-01929-6.

## Background

Crohn’s disease (CD) and ulcerative colitis (UC) as the main entities of inflammatory bowel disease (IBD) are documented with rising prevalence in western world populations [1–4]. Genetic, immunologic and environmental factors are involved in IBD pathogenesis, which is characterized by chronic recurrent inflammation of the intestinal mucosa [5].

Fecal metabolites are emerging as biomarkers in clinical management of patients with IBD. Fecal calprotectin is routinely used to screen for intestinal inflammation and to predict disease relapse [6, 7]. Calprotectin is increased with any intestinal inflammation [8, 9], and disease-specific biomarkers are warranted for diagnosis and follow-up of patients with IBD [10, 11].

The gut microbiome has gained substantial attention over the last decades [12], and genetic, disease-related, and environmental factors are thought to contribute to reduced diversity of the gut microbiota in IBD [8, 13].

Short chain fatty acids (SCFA) such as acetate, propionate, butyrate, and isobutyrate are derived from microbial fermentation of dietary fibers, and their fecal levels have been assessed in patients with IBD [14]. At this time, data are not consistent and there are reports of reduced as well as unchanged fecal SCFA levels of patients with IBD in comparison to healthy controls [15–19]. A meta-analysis uncovered a decline of fecal acetate and butyrate levels of CD patients, lower fecal actetate of patients with active UC, and higher fecal butyrate of UC patients in remission, and all of these patient cohorts were compared to healthy controls [13]. Fecal butyrate levels of children with IBD were found increased in comparison to controls [17].

Primary sclerosing cholangitis (PSC) is a rare cholestatic disease closely associated with IBD, and about 70% of PSC patients have IBD (PSC-IBD) [20]. Although the pathogenic mechanisms of PSC-IBD are still unknown, gut dysbiosis and disturbed bile acid metabolism seem to have a role herein [20]. Various studies proved a protective function of SCFAs for liver health [21] but fecal SCFA levels of PSC-IBD patients have not been measured as far as we know. PSC diagnosis is still a challenge and non-invasive biomarkers for monitoring of PSC development in IBD are urgently needed [22–24].

Urine as a body fluid has been used for diagnostic purposes for a long time [25–27]. Indol is produced by bacterial processing of dietary L-tryptophan, and is further processed by the liver to its major conjugate 3-indoxyl sulfate, which is then urinary excreted [28, 29]. Accordingly, low urinary 3-indoxyl sulfate concentrations are considered biomarkers for a disturbed gut microbiome [30]. One study found an increase of urinary 3-indoxyl sulfate levels of patients with active CD, who achieved clinical response [31] pointing to reduced levels in active disease. Another study observed elevated urinary 3-indoxyl sulfate levels of IBD patients compared to healthy controls [32].

The main aim of this study was the analysis of fecal SCFA levels of patients with IBD and patients with PSC-IBD to find out whether analysis of these metabolites is of diagnostic value. Urinary 3-indoxylsulfate of IBD patients was measured to clarify its role as a disease biomarker.

## Materials and methods

### Patients

Patients with an established diagnosis of IBD were recruited from the outpatient and inpatient clinic at the Department of Internal Medicine I (University Hospital of Regensburg) from December 6, 2021, to January 31, 2023. IBD diagnosis was based on histologic, endoscopic, and clinical criteria [33, 34]. PSC-IBD was diagnosed based on histologic, endoscopic, and clinical criteria [35]. Patients with known coagulopathy were excluded from the study. All patients coming to our clinic were asked whether they like to participate in the study, and all patients, who agreed to participate, were included. Feces of 64 patients and urine of 45 of these patients were available for this study. These patients were all treated with appropriate medications such as mesalazin, azathioprine, glucocorticoids and/or biologics. The time points for start of the first therapy were documented for 40 patients and was 11 (3—20) years.

Moreover, feces of 17 healthy controls and urine of 13 of these healthy controls were collected and analyzed. These healthy controls were hospital staff members, students, and partners of the patients. Controls lived in the same area as the patients. There was a large age spread among the patients, and young as well as elderly controls were enrolled. Most of the controls asked did not agree to provide stool samples, and all controls willing to provide samples were included. The study groups are described in Table 1.

**Table 1:** Characteristics of patients and controls. Urine was from the same patient / healthy control, of whom feces was collected. Data are given as median and interquartile range. There were no significant differences between these groups. Unpaired Student´s t-test was used for comparison of patients / controls for SCFA and 3-indoxylsulfate analysis. Mann Whitney U-test was used for comparison of the respective patient and control cohorts. Chi-square test was used for categorical variables and *P* > 0.05 was obtained for all of these comparisons

Characteristics	Patients for SCFA analysis	Patients for 3-indoxyl sulfate analysis	Controls for SCFA analysis	Controls for 3-indoxyl sulfate analysis
Number (females/males)	64 (29/35)	45 (18/27)	17 (11/6)	13 (9/4)
Age (years)	48 (34–54)	42 (32–53)	48 (26–58)	42 (25–56)
BMI (kg/m^2^)	24.2 (22.1–28.1)	24.2 (22.0–27.1)	n.d	n.d
CRP (mg/L)	1.9 (0.8–8.4)	2.3 (0.9 -11.8)	n.d	n.d
Creatinine (mg/dL)	0.84 (0.73–0.90)	0.84 (0.76–0.89)	n.d	n.d
GFR (mL/min)	99 (91–110)	100 (90–110)	n.d	n.d

*BMI* Body mass index, *CRP* C-reactive protein, *GFR* Glomerular filtration rate, *n.d.* Not documented

### Measurement of C-reactive protein, serum creatinine and fecal calprotectin, and calculation of GFR

CRP was determined by a particle-enhanced immunoturbidimetric assay using the Cobas Pro C analyzer and the appropriate assays (Roche, Penzberg, Germany). The lower detection limit of this measurement is 0.6 mg/L. Because the CRP-test and not the hsCRP test, which is also distributed by this company, has been used, we refer to CRP in the manuscript.

The measurement of serum creatinine was performed using Cobas Pro C from Roche. The enzymatic method for the determination of serum creatinine was carried out by the conversion of creatinine by creatininase, creatinase and sarcosine oxidase to glycine, formaldehyde, and hydrogen peroxide. The released hydrogen peroxide is used by peroxidase to form a quinoneiminine dye using 4-aminophenazone and HTIBa as substrate. The color intensity of the quinoneiminine dye is directly proportional to the creatinine concentration in the reaction mixture.

For the calculation of GFR the equation described by Levey et al. [36] was used. The Chronic Kidney Disease Epidemiology Collaboration creatinine equation is: GFR = 141 × min (serum creatinine /κ, 1)α × max(serum creatinine /κ, 1)-1.209 × 0.993age × 1.018 [if female], where κ is 0.7 for females and 0.9 for males, α is -0.329 for females and -0.411 for males [36].

For the measurement of fecal calprotectin, a sandwich immunoassay was performed, in which paramagnetic beads are coated with calprotectin-specific capture antibodies (QUANTA Flash® Calprotectin Reagents, Inova Diagnostics; San Diego, CA, USA). The analysis is performed using the BIO-FLASH chemiluminescence analyzer (Inova Diagnostics, Werfen, Austria).

### Analysis of urinary 3-indoxyl sulfate and urinary creatinine

Urinary 3-indoxyl sulfate levels were analysed by means of reversed-phase liquid chromatography-electrospray ionization-tandem mass spectrometry, as described elsewhere [37]. Urinary creatinine was determined by the creatinine parameter assay kit as indicated by the supplier (R&D Systems, Wiesbaden-Nordenstadt, Germany).

### Preparation of feces for lipid analysis

The fecal samples were collected by the patients / controls in tubes containing 70% isopropanol. Addition of isopropanol was shown to preserve fecal SCFA levels [38]. The fecal human samples were homogenized in a gentleMACS™ Dissociator (Miltenyi Biotec GmbH, Bergisch Gladbach, Germany) in 70% isopropanol. An aliquot was dried overnight in a vacuum centrifuge to determine the dry weight of the samples. The homogenates were diluted to a concentration of 2.0 mg dry weight/mL. The samples were stored at -80 °C until use and were kept on ice during processing.

### Analysis of acetate, propionate, butyrate and isobutyrate

For SCFA measurement, aliquots of the homogenates described above were centrifuged and derivatized to 3-nitrophenylhydrazones. SCFAs were quantified by liquid chromatography with tandem mass spectrometry (LC–MS/MS). A Kinetex®2.6 µm XB-C18, 50 × 2.1 mm (Phenomenex, Torrance, CA, USA) was used for SCFA separation. Water containing 0.1% formic acid was used as mobile phase A and acetonitrile containing 0.1% formic acid as mobile phase B. The elution was started with 10% B, which was linearly increased to 20% B until 0.3 min, and to 23% B at 2.5 min. The column was washed with 100% B and was reequilibrated with 90% A. 2 µl of the sample was injected and analysed with a column flow of 500 µL at 60 °C. The following internal standards were added prior derivatization [13C,D3]-FA 2:0, [D5]-FA 3:0 and [D7]-FA 4:0. Quantification was performed using matrix-based calibration lines generated with authentic standards [38].

### Statistical analysis

Data are shown as boxplots or bar charts (± standard deviation), and Mann Whitney U-test, Kruskal–Wallis Test and Spearman correlation were used as statistical tests (SPSS Statistics 26.0 program, IBM, Leibniz Rechenzentrum, München. Germany). Continuous variables in the tables and the text of the manuscript are given as median and interquartile range. The Chi-square test was performed for categorical variables and an unpaired t-test for paired data (Ms Excel 2016). A value of *P* < 0.05 was regarded as significant.

## Results

### Fecal SCFA and urinary 3-indoxyl sulfate levels of IBD patients and healthy controls

**Fig. 1:**
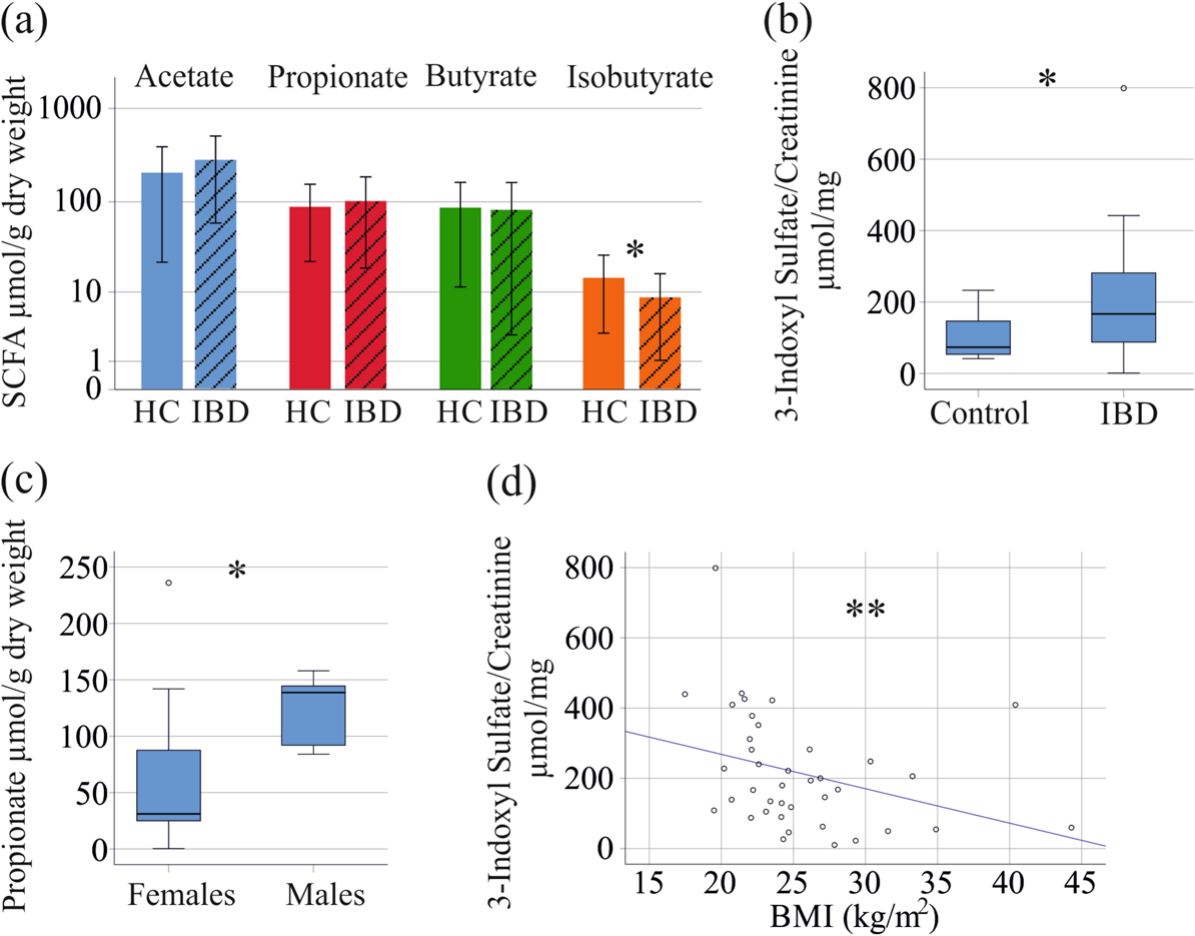
Fecal SCFAs and urinary 3-indoxyl sulfate levels of healthy controls and patients with IBD. **a** SCFAs in stool of healthy controls (HC) and patients with IBD (IBD); **b** Urinary 3-indoxyl sulfate levels of controls and patients with IBD; **c** Fecal propionate levels of female and male controls; **d** Correlation of urinary 3-indoxyl sulfate levels with BMI in the IBD cohort. Spearman correlation coefficient r =—0.472. Small circles in b and c mark outliers. * *p* < 0.05

Fecal acetate, propionate and butyrate levels were similar between the 17 controls and the 64 patients with IBD. Isobutyrate in the stool of the patients was lower in comparison to the healthy controls (*P* = 0.035; Fig. 1a). Urine of 45 patients and 13 controls, which was from the same patients where feces was analyzed, was available for this study. The IBD subgroup for 3-indoxyl sulfate analysis had similar sex distribution, age, body mass index (BMI), C-reactive protein (CRP), creatinine, and glomerular filtration rate (GFR) in comparison to the whole cohort (Table 1). Urinary 3-indoxyl sulfate normalized to urinary creatinine of the 45 patients was higher compared to the 13 controls (*P* = 0.030; Fig. 1b).

Sex-based differences of fecal SCFAs have been described before [39], and in our control cohort fecal propionate of the 6 males was higher in comparison to the 11 females (*P* = 0.011; Fig. 1c). Concentration of urinary 3-indoxyl sulfate, fecal acetate, butyrate and isobutyrate did not differ between sexes. None of these metabolites correlated with age (*P* > 0.05 for all).

In the IBD cohort, the 29 females had similar concentrations of fecal acetate, propionate, butyrate and isobutyrate as the 35 males. Urinary 3-indoxyl sulfate normalized to urinary creatinine did not differ between sexes. Because these metabolites of male and female patients were similar, and a sex-specific effect in the control cohort was only noticed for propionate, which did not differ between patients and controls, we did not perform sex-specific analysis in our patient cohort. None of the metabolites measured correlated with age of the IBD patients (*P* > 0.05 for all).

Fecal SCFA levels of IBD patients did not correlate with BMI whereas urinary 3-indoxyl sulfate of IBD patients was negatively correlated with BMI (r =—0.472, *P* = 0.002; Fig. 1d).

It has to be noted that fecal SCFAs of IBD patients correlated with each other but not with levels of urinary 3-indoxyl sulfate (Table 2).

**Table 2:** Spearman correlation coefficients of fecal SCFA and urinary 3-indoxyl sulfate levels (normalized to urinary creatinine) of patients with IBD. * *P* < 0.05, *** *P* < 0.001

Metabolite	Acetate	Propionate	Butyrate	Isobutyrate
Acetate	-	0.743^***^	0.793^***^	0.317^*^
Propionate	0.743^***^	-	0.701^***^	0.602^***^
Butyrate	0.793^***^	0.701^***^	-	0.496^***^
Isobutyrate	0.317^*^	0.602^***^	0.496^***^	-
3-Indoxyl Sulfate	-0.166	-0.265	-0.118	-0.111

### Fecal SCFA and urinary 3-indoxyl sulfate levels of CD and UC patients and correlation with laboratory values and fecal calprotectin

**Fig. 2:**
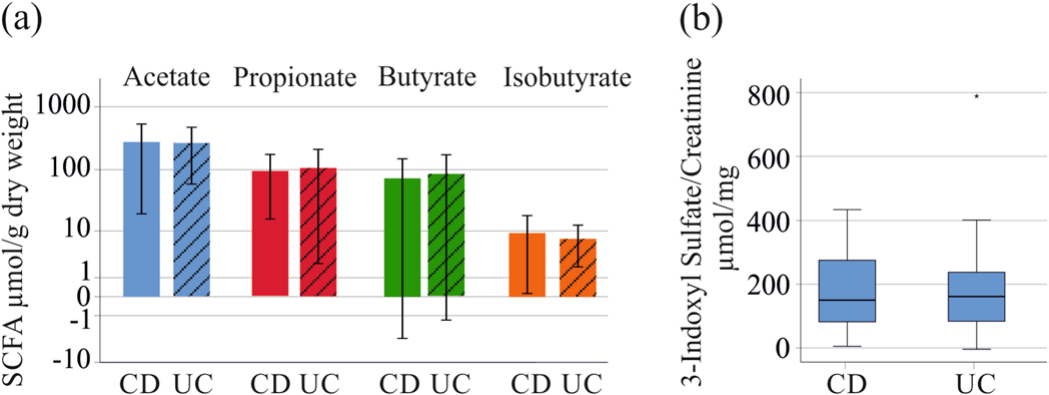
Fecal SCFA levels and urinary 3-indoxyl sulfate levels of patients with CD and UC. **a** SCFAs in stool of CD and UC patients; **b** Urinary 3-indoxyl sulfate levels of CD and UC patients. The asterisk in b indicates an outlier

The 43 CD patients and the 21 UC patients did not differ with regard to age, BMI, CRP, creatinine, GFR and fecal calprotectin levels (Table S1). Fecal concentrations of the SCFAs and urinary 3-indoxyl sulfate levels were similar between CD and UC patients (Fig. 2a, b; Table S2).

The concentration of urinary 3-indoxyl sulfate of CD (*P* = 0.039) and UC (*P* = 0.075) patients was higher in contrast to the healthy controls (Table S2). Fecal isobutyrate levels of CD patients (*P* = 0.069) as well as UC patients (*P* = 0.036) were reduced in comparison to healthy controls (Table S2).

In the IBD cohort fecal propionate levels positively correlated with CRP (*P* = 0.022; Table 3). No other correlations between fecal SCFAs and urinary 3-indoxyl sulfate with CRP, serum creatinine, GFR or fecal calprotectin were detected (Table 3). In the CD subgroup no significant correlations were found. In UC patients, CRP and fecal calprotectin positively correlated with fecal acetate levels (*P* = 0.024 and *P* = 0.040, respectively) and fecal butyrate levels (*P* = 0.025 and *P* = 0.005, respectively). There was a positive correlation of fecal propionate and CRP in UC (*P* = 0.042; Table 3).

**Table 3:** Spearman correlation coefficients for the correlation of 3-indoxyl sulfate and short-chain fatty acid levels with CRP, creatinine, GFR and fecal calprotectin. Analysis was performed using data of all IBD patients, of CD patients and of UC patients. Significant correlations are in bold. * *P* < 0.05, ** *P* < 0.01

Marker	3-Indoxyl Sulfate/Creatinine	Acetate	Propionate	Butyrate	Isobutyrate
**Patients with IBD**					
CRP	-0.064	0.232	0.297*	0.217	0.113
Creatinine	-0.264	-0.016	-0.141	-0.033	-0.161
GFR	0.022	0.166	0.148	0.145	0.163
Fecal calprotectin	0.162	0.137	0.118	0.094	0.082
**Patients with Crohn´s disease**					
CRP	-0.130	0.061	0.169	0.031	0.089
Creatinine	-0.156	0.051	-0.074	0.063	-0.067
GFR	-0.206	0.124	0.095	0.029	0.110
Fecal calprotectin	0.151	-0.006	-0.052	-0.151	-0.043
**Patients with Ulcerative colitis**					
CRP	-0.093	0.528*	0.471*	0.511*	0.016
Creatinine	-0.414	-0.089	-0.220	-0.087	-0.120
GFR	0.458	0.209	0.169	0.245	0.106
Fecal calprotectin	0.254	0.474*	0.414	0.602**	0.353

*CRP* C-reactive protein, *GFR* Glomerular filtration rate

**Fig. 3:**
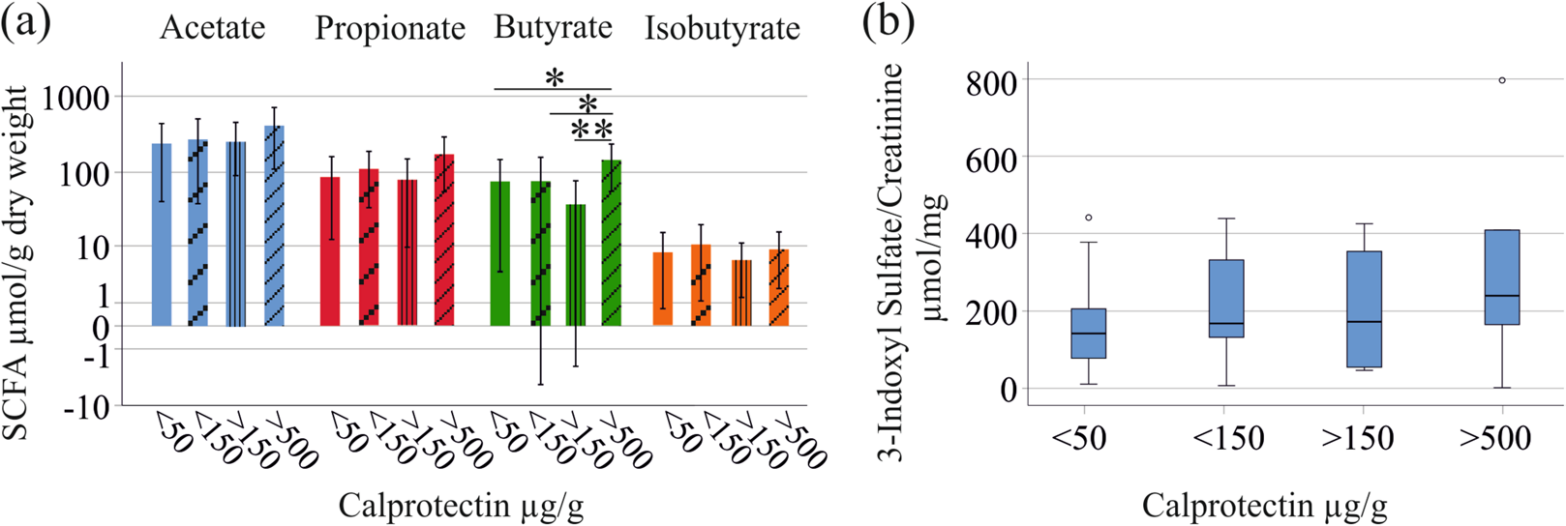
Fecal SCFA levels and urinary 3-indoxyl sulfate levels of patients with IBD stratified for fecal calprotectin levels. **a** SCFAs in stool of IBD patients with fecal calprotectin < 50 µg/g, < 150 µg/g, > 150 µg/g and > 500 µg/; **b** Urinary 3-indoxyl sulfate of patients with IBD stratified for fecal calprotectin levels. The small circles in b indicate outliers. * *p* < 0.05, ** *p* < 0.01

 Next, distribution of SCFAs was analyzed in the patient cohort categorized according to fecal calprotectin levels. Twenty-nine patients had calprotectin levels below 50 µg/g, 17 patients had levels from 50—149 µg/g, 8 patients from 150—500 µg/g, and 7 patients > 500 µg/g (values of 3 patients were unknown). Butyrate was significantly higher in this latter group in comparison to the three other subgroups (*P* = 0.016, 0.023 and 0.003 for comparison of < 50 and > 500, < 150 and > 500, and > 150 and > 500 µg/g calprotectin, respectively). Fecal acetate, propionate, isobutyrate, and urinary 3-indoxyl sulfate levels did not change with higher fecal calprotectin levels (Fig. 3a, b).

**Fig. 4:**
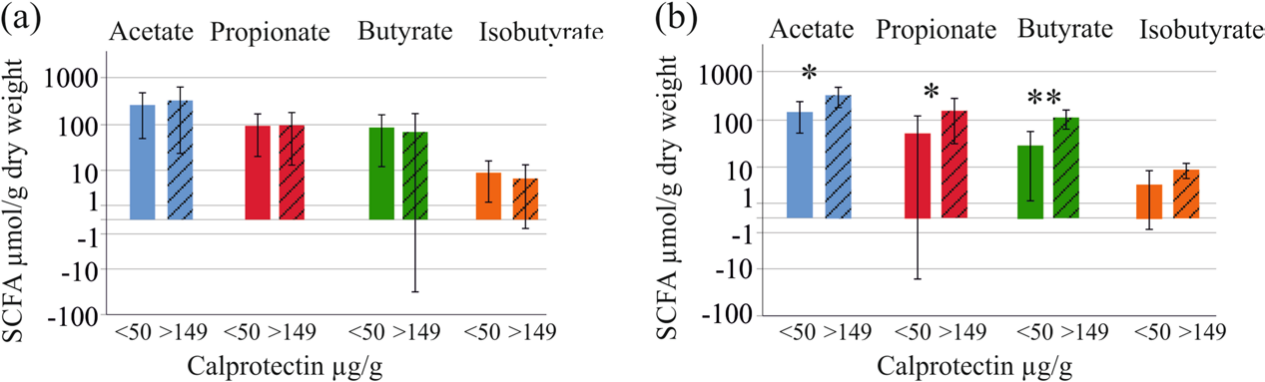
Fecal SCFA levels of patients with CD and UC with low and high calprotectin levels. **a** Fecal SCFA levels of CD patients with calprotectin levels below 50 µg/g (22 patients) and patients with levels from 150 to > 500 µg/g (9 patients); **b** Fecal SCFA levels of UC patients with calprotectin levels below 50 µg/g (7 patients) and patients with levels from 150 to > 500 µg/g (6 patients). * *p* < 0.05, ** *p* < 0.01

The number of patients with high calprotectin levels was too low for a separate analysis of UC and CD patients. Comparison of UC patients with calprotectin levels < 50 µg/g (7 patients) and patients with levels from 150 to > 500 µg/g (6 patients) showed that fecal acetate (*P* = 0.016), propionate (*P* = 0.046) and butyrate (*P* = 0.007) of the patients with higher calprotectin were increased. No such differences were observed in the CD patients where 22 patients had fecal calprotectin < 50 µg/g and 9 patients levels from 150 to > 500 µg/g (Fig. 4a, b).

### Urinary 3-indoxyl sulfate and fecal SCFAs in relation to extraintestinal manifestations and fistulas

Urinary 3-indoxyl sulfate levels of the 21 patients with extra-intestinal manifestations from IBD and the 8 patients with fistulas did not differ from patients without these complications. SCFA levels of the 32 patients who suffered from extra-intestinal manifestations and the 15 patients with fistulas did not differ from patients without extra-intestinal manifestations or patients without fistulas (data not shown).

### Fecal SCFA levels of PSC-IBD patients

PSC is a rare disease and 20 PSC-IBD patients were included. Patients with PSC-IBD had higher serum levels of aspartate aminotransferase (AST, *P* = 0.048), alkaline phosphatase (AP, *P* = 0.002) and bilirubin (*P* = 0.004) in comparison to IBD patients not suffering from PSC (Table S3). Age, BMI, alanine aminotransferase (ALT), gamma glutamyltransferase, CRP, creatinine, GFR and fecal calprotectin did not differ among these patient cohorts (Table S3).

**Fig. 5:**
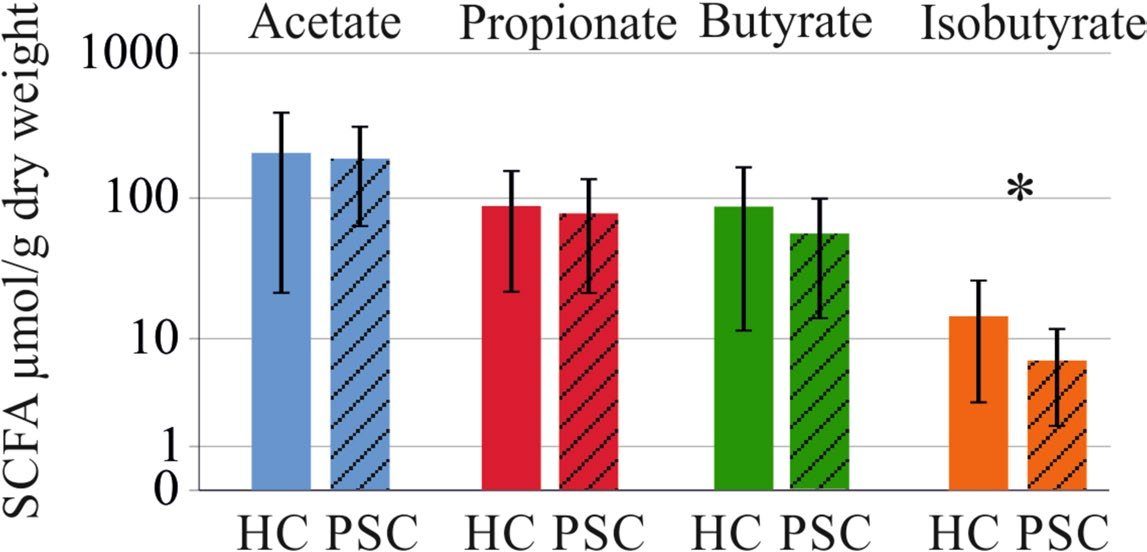
Fecal SCFAs of healthy controls (HC) and patients with PSC-IBD (PSC) * *p* < 0.05

SCFAs did not differ between IBD and PSC-IBD (-*P* > 0.05 for all). Fecal isobutyrate was lower in PSC-IBD compared to controls (*P* = 0.028) (Fig. 5).

In the PSC-IBD cohort butyrate was positively correlated with AST (*P* = 0.040) and bilirubin (*P* = 0.018) (Table 4).

**Table 4:** Spearman correlation coefficients of SCFA levels and clinical disease measures of patients with PSC-IBD. Significant correlations are given in bold. * *P* < 0.05

	Acetate	Propionate	Butyrate	Isobutyrate
AST (U/L)	0.489	0.260	0.553^*^	0.044
ALT (U/L)	0.370	0.251	0.372	0.119
GammaGT (U/L)	0.161	0.211	0.383	0.066
AP (U/L)	0.244	0.235	0.367	-0.011
Bilirubin (mg/dL)	0.529	0.217	0.619^*^	-0.055
CRP (mg/L)	0.084	-0.038	-0.043	-0.330
Calprotectin (µg/g)	0.202	0.454	0.187	0.019

Alanine aminotransferase: *ALT* Alkaline phosphatase, *AP* Aspartate aminotransferase, *AST*, C-reactive protein, *CRP* Gamma-glutamyltransferase: gammaGT

## Discussion

Low levels of fecal SCFAs as well as urinary 3-indoxyl sulfate were suggested to reflect a decreased microbiome diversity [13, 30, 40]. Present analysis detected lower isobutyrate levels in stool and higher 3-indoxyl sulfate levels in urine of patients with IBD in comparison to healthy controls. This study shows that the decreased microbiome diversity commonly described in IBD [8, 13, 41–45] is not very well reflected by these metabolites. This suggestion is in accordance with the study by Sze et al., which was unable to identify associations between the fecal bacterial community structure and SCFA levels [46]. Reduction of butyrate producing bacteria in UC patients was not related to a decline of fecal butyrate, a finding also supporting this conclusion [19].

Higher urinary 3-indoxyl sulfate levels of IBD patients compared to healthy controls have been reported before [32]. In the current cohort, urinary 3-indoxyl sulfate of IBD patients was increased compared to healthy controls. Urinary 3-indoxyl sulfate levels were not related to clinical markers of inflammation and could not discriminate UC from CD patients.

Serum creatinine and GFR as markers of renal function [47] did not correlate with 3-indoxyl sulfate levels in urine excluding a close relationship between renal excretion of this metabolite and kidney function.

Interestingly, urinary 3-indoxyl sulfate levels negatively correlated with BMI in the IBD cohort. Lower microbiome diversity in obesity has been reported in some studies, but findings published so far are quite heterogeneous [48]. Thus, it is possible that the controls and IBD patients have different BMI, which was not documented for the controls in our study, and this may contribute to different levels of 3-indoxyl sulfate between patients and controls. It should be noted that fecal SCFAs did not correlate with BMI of the IBD cohort, and urinary 3-indoxyl sulfate may be a more sensitive marker for obesity-related changes of the gut microbiome.

Only few studies have investigated sex-specific differences of urinary and fecal metabolites. Urinary 3-indoxyl sulfate concentrations were similar between sexes. However, healthy males had higher fecal propionate than healthy females in accordance with a recent study [39]. Such a sex-specific difference was not detected in the IBD cohort for a so far unknown reason.

Fecal SCFA levels have been analyzed before [13], and the current study cohort is larger than most of these previous ones. Moreover, isobutyrate in feces has not been studied in very depth so far. Of the four fecal SCFAs analysed, isobutyrate was lower in stool of IBD patients in comparison to controls. Such differences were also found for the comparison of controls and UC or CD patients, who had similar levels of fecal SCFAs.

Patients from North India with active UC had lower butyrate, isobutyrate and acetate when compared to healthy controls. These metabolites did not differ among patients with active disease and patients in remission [18]. In the cohort analysed in our study, fecal isobutyrate did not change with higher fecal calprotectin levels as an established marker of active disease and is, therefore, not related to disease activity.

Fecal propionate weakly and positively correlated with serum CRP levels indicating an association with systemic inflammation. Fecal propionate did, however, not correlate with fecal calprotectin levels, and did not differ between patients with IBD and controls. Fecal acetate did not change in IBD and was not associated with disease severity.

CD and UC are the main IBD entities and differ in disease features [44, 49]. Acetate, propionate and butyrate positively correlated with serum CRP of UC patients, which is a useful marker to monitor disease activity in IBD [50]. Acetate and butyrate positively correlated with fecal calprotectin levels of UC patients and acetate, propionate and butyrate were accordingly higher in stool of UC patients with increased levels of fecal calprotectin, a well accepted marker of disease severity [6, 7, 51, 52]. No such associations were observed in the CD cohort. This observational study can, however, not provide any explanation for these differences between CD and UC. The two cohorts had similar age, BMI, CRP and fecal calprotectin levels excluding these markers as confounding factors.

Of note, in the whole cohort fecal butyrate levels were found to be modestly increased in patients with high fecal calprotectin levels in contrast to patients with lower calprotectin levels. This finding is in contrast to a previous study showing lower fecal butyrate in active IBD [15]. Higher fecal butyrate levels were, however, identified in children with IBD [17] and UC patients in remission in comparison to healthy controls [13]. Elevated SCFA levels were related to gut microbiome dysbiosis, increased gut permeability and higher levels of high-sensitive CRP levels in blood [53].

Butyrate was shown to reduce inflammation of dendritic cells localized in the intestinal mucosa. Butyrate, moreover, increases interleukin-10 levels, and this cytokine subsequently activates anti-inflammatory regulatory T-cells [54]. Higher levels of butyrate may thus be considered as protective in the severely inflamed gut of IBD patients.

Notably, fecal levels of acetate, propionate, butyrate, and isobutyrate were strongly and positively correlated to each other pointing to common regulatory pathways. These may be factors affecting the composition of the gut microbiome, or pathways regulating their absorption by monocarboxylate transporters expressed in colonocytes [55].

SCFAs produced by bacteria are either excreted in feces or enterically absorbed [56]. Higher fecal levels may thus be the result of increased production and/or impaired absorption in the colon. Hence, it was also shown that higher, rather than lower, fecal SCFA excretion was related to gut dysbiosis [53]. Accordingly, more research is needed to clarify the pathological role of fecal SCFAs.

PSC-IBD is a disease closely related to IBD [57, 58]. Fecal SCFA levels between IBD and PSC-IBD patients were similar, and lower isobutyrate of PSC-IBD patients in contrast to controls is related to IBD. Thus, PSC-IBD development in IBD can not be diagnosed by analysis of fecal SCFAs.

### Strengths and limitations of the study

The strength of our study includes the measurement of fecal SCFAs and urinary 3-indoxyl sulfate of the identical patients. An additional strength is that fecal samples of patients with PSC-IBD were analyzed, which has not been done before as far as we know. Moreover, our cohort was large enough for separate analysis of CD and UC patients. We also have to acknowledge limitations to our study. This is a single center cohort study and diet was not assessed. Furthermore, composition of the gut microbiome was not analyzed in this study. The number of controls was low and BMI as well as laboratory measures of this cohort were not documented. There were only seven patients, who had high fecal calprotectin levels and associations of SCFA levels with IBD disease activity needs further study.

## Conclusions

IBD is associated with increased urinary 3-indoxyl sulfate concentrations and lower levels of fecal isobutyrate in comparison to healthy controls. Modest positive associations of fecal acetate and butyrate with fecal calprotectin were observed in patients with UC but not CD. These metabolites could not discriminate CD and UC, and did not show added value in monitoring disease activity.

### Supplementary Information

**Additional file 1: Table S1. **Characteristics of Crohn´s disease (CD) and ulcerative colitis (UC) patients, where feces was available for SCFA analysis, and of the same patients, where urine was available for 3-indoxyl sulfate analysis. Median values and interquartile ranges are given.  Unpaired Student´s t-test was used for comparison of patients / controls for SCFA and 3-indoxylsulfate analysis. Mann Whitney U-test was used for comparison of the respective patient and control cohorts. Chi-square test was used for categorical variables. There were no significant differences between these groups. **Table S2. **Median concentrations and interquartile range of urinary 3-indoxyl sulfate and fecal SCFA levels of controls, Crohn´s disease and ulcerative colitis patients. Pairwise comparison of control and CD patients as well as controls and UC patients were performed. * *P* < 0.05. **Table S3. **Characteristics of IBD and PSC-IBD patients as well as the controls where feces was available for SCFA analysis. Median value and interquartile range is given in the table.

## Data Availability

The datasets used and/or analysed during the current study are available from the corresponding author on reasonable request.

## Abbreviations

ALT: Alanine aminotransferase
AP: Alkaline phosphatase
AST: Aspartate aminotransferase
CD: Crohn´s Disease
CRP: C-reactive protein
IBD: Inflammatory Bowel Disease
PSC: Primary sclerosing cholangitis
SCFA: Short chain fatty acids
UC: Ulcerative colitis
