# Supplementary material for: Fecal short chain fatty acids and urinary 3-indoxyl sulfate do not discriminate between patients with Crohn´s disease and ulcerative colitis and are not of diagnostic utility for predicting disease severity
Source: Lipids Health Dis. 2023 Oct 3;22:164. doi: 10.1186/s12944-023-01929-6 (PMC10546683; doi:10.1186/s12944-023-01929-6)
Supplement: Supplementary file 1 — Additional file 1: Table S1. Characteristics of Crohn´s disease (CD) and ulcerative colitis (UC) patients, where feces was available for SCFA analysis, and of the same patients, where urine was available for 3-indoxyl sulfate analysis. Median values and interquartile ranges are given. Unpaired Student´s t-test was used for comparison of patients / controls for SCFA and 3-indoxylsulfate analysis. Mann Whitney U-test was used for comparison of the respective patient and control cohorts. Chi-square test was used for categorical variables. There were no significant differences between these groups. Table S2. Median concentrations and interquartile range of urinary 3-indoxyl sulfate and fecal SCFA levels of controls, Crohn´s disease and ulcerative colitis patients. Pairwise comparison of control and CD patients as well as controls and UC patients were performed. * P < 0.05. Table S3. Characteristics of IBD and PSC-IBD patients as well as the controls where feces was available for SCFA analysis. Median value and interquartile range is given in the table. [file 12944_2023_1929_MOESM1_ESM.docx]

Supplementary data

Fecal short chain fatty acids and urinary 3-indoxyl sulfate do not discriminate between patients with Crohn´s disease and ulcerative colitis and are not of diagnostic utility for predicting disease severity

Hauke Christian Tews ^1^, Tanja Elger ^1^, Stefan Gunawan ^1^, Tanja Fererberger ^1^, Stefanie Sommersberger ^1^, Johanna Loibl ^1^, Muriel Huss ^1^, Gerhard Liebisch ^2^, Martina Müller ^1^, Arne Kandulski ^1^ and Christa Buechler ^1^ *

**Table S1.** Characteristics of Crohn´s disease (CD) and ulcerative colitis (UC) patients, where feces was available for SCFA analysis, and of the same patients, where urine was available for 3-indoxyl sulfate analysis. Median values and interquartile ranges are given. Unpaired Student´s t-test was used for comparison of patients / controls for SCFA and 3-indoxylsulfate analysis. Mann Whitney U-test was used for comparison of the respective patient and control cohorts. Chi-square test was used for categorical variables. There were no significant differences between these groups.

| Characteristics | CD Patients for SCFA analysis | CD Patients for 3-indoxyl sulfate analysis | UC Patients for SCFA analysis | UC Patients for 3-indoxyl sulfate analysis |
| --- | --- | --- | --- | --- |
| Number (females/males) | 43 (22/21) | 30 (15/15) | 21 (7/14) | 15 (3/12) |
| Age (years) | 43 (34-53) | 42 (33-52) | 50 (29-57) | 48 (28-56) |
| BMI (kg/m^2^) | 24.2 (22.1-27.1) | 24.2 (21.7-27.0) | 23.8 (22.0-31.3) | 24.2 (22.2-30.4) |
| CRP (mg/L) | 2.3 (1.0-8.6) | 3.7 (1.1-11.5) | 1.8 (0.6-12.1) | 1.6 (0.6-20.6) |
| Creatinine (mg/dL) | 0.81 (0.73-0.89) | 0.80 (0.75-0.89) | 0.86 (0.79-0.99) | 0.85 (0.79-1.00) |
| GFR (mL/min) | 100 (91-111) | 100 (75-120) | 98 (85-107) | 98 (85-107) |
| Fecal calprotectin µg/g | 44 (29-122) | 44 (31-143) | 65 (34-259) | 73 (34-883) |

Body mass index: BMI; C-reactive protein: CRP; Glomerular filtration rate: GFR

**Table S2.** Median concentrations and interquartile range of urinary 3-indoxyl sulfate and fecal SCFA levels of controls, Crohn´s disease and ulcerative colitis patients. Pairwise comparison of control and CD patients as well as controls and UC patients were performed. * *P* < 0.05.

| Metabolite | Controls | Crohn´s disease | Ulcerative colitis |
| --- | --- | --- | --- |
| 3-Indoxyl Sulfate/Creatinine µmol/mg | 73.6 (52.9-151.6)* | 156.2 (81.4-299.6)* | 167.4 (77.5-248.1) |
|  |  |  |  |
| Acetate µmol/g | 205.5 (84.3-266.0) | 221.5 (147.0-307.0) | 223.5 (147.9-405.1) |
| Propionate µmol/g | 86.00 (28.1-141.8) | 89.5 (27.2-138.5) | 92.8 (14.1-170.3) |
| Butyrate µmol/g | 80.0 (28.9-111.0) | 62.0 (63.5-97.0) | 69.8 (22.6-114.1) |
| Isobutyrate µmol/g | 14.0 (5.9-22.4)* | 6.7 (3.9-11.3) | 7.2 (1.4-12.3)* |

**Table S3.** Characteristics of IBD and PSC-IBD patients as well as the controls where feces was available for SCFA analysis. Median value and interquartile range is given in the table. Mann Whitney U-test was used for comparison of the cohorts. Chi-square test was used for categorical variables. * p < 0.05, ** p < 0.001.

Alanine aminotransferase: ALT; alkaline phosphatase: AP; aspartate aminotransferase: AST; Body mass index: BMI; C-reactive protein: CRP; gamma glutamyltransferase: gammaGT; Glomerular filtration rate: GFR; Not documented: n.d.

| Characteristics | IBD patients for SCFA analysis | PSC-IBD Patients for SCFA analysis | Controls for SCFA analysis |
| --- | --- | --- | --- |
| Number (females/males) | 64 (29/35) | 20 (6/14) | 17 (11/6) |
| Age (years) | 48 (34-54) | 47 (34-55) | 48 (26-58) |
| BMI (kg/m^2^) | 24.2 (22.1-28.1) | 24.4 (18.1-29.9) | n.d. |
| CRP (mg/L) | 1.9 (0.8-8.4) | 3.1 (1.7-16.2) | n.d. |
| Creatinine (mg/dL) | 0.83 (0.73-0.90) | 0.81 (0.66-1.04) | n.d. |
| GFR (mL/min) | 99 (91-110) | 96 (71-104) | n.d. |
| Fecal calprotectin µg/g | 55 (33-143) | 38 (18-184) | n.d. |
| AST | 25 (21-28)* | 36 (21-69)* | n.d. |
| ALT | 20 (17-28) | 37 (17-61) | n.d. |
| GammaGT | 25 (18-33) | 29 (22-185) | n.d. |
| AP | 69 (54-87) | 109 (71-207)** | n.d. |
| Bilirubin | 0.4 (0.4-0.6) | 0.7 (0.5-1.1)** | n.d. |
